# DNA hypermethylation of *sirtuin 1* (*SIRT1*) caused by betel quid chewing—a possible predictive biomarker for malignant transformation

**DOI:** 10.1186/s13148-019-0806-y

**Published:** 2020-01-13

**Authors:** Shajedul Islam, Osamu Uehara, Hirofumi Matsuoka, Yasuhiro Kuramitsu, Bhoj Raj Adhikari, Daichi Hiraki, Seiko Toraya, Asiri Jayawardena, Ichiro Saito, Malsantha Muthumala, Hiroki Nagayasu, Yoshihiro Abiko, Itsuo Chiba

**Affiliations:** 1 Division of Disease Control and Molecular Epidemiology, Department of Oral Growth and Development, School of Dentistry, Health Sciences University of Hokkaido, Hokkaido, 061-0293 Japan; 2 Division of Oral Medicine and Pathology, Department of Human Biology and Pathophysiology, School of Dentistry, Health Sciences University of Hokkaido, Hokkaido, 061-0293 Japan; 3 Research Institute of Cancer Prevention, Health Sciences University of Hokkaido, Hokkaido, 061-0293 Japan; 4 Department of General Education, School of Dental Medicine, Tsurumi University, Kanagawa, 230-8501 Japan; 5 Department of Pathology, School of Dental Medicine, Tsurumi University, Kanagawa, 230-8501 Japan; 6 Department of Oral and Maxillofacial Surgery, Army Hospital, Colombo, Sri Lanka; 7 Division of Oral and Maxillofacial Surgery, Department of Human Biology and Pathophysiology, School of Dentistry, Health Sciences University of Hokkaido, Hokkaido, 061-0293 Japan

**Keywords:** Oral cancer, Sirtuin 1, Betel quid chewing, Arecoline, Malignant transformation

## Abstract

**Background:**

DNA hypermethylation of tumor suppressor genes is observed in precancerous lesions and oral cancer of individuals with the habits of betel quid (BQ) chewing. *SIRT1* has been identified as playing a role in the maintenance of epithelial integrity, and its alteration is often related to carcinogenesis. However, the methylation and transcription status of *SIRT1* in patients with BQ chewing-related oral cancer has not been investigated. We examined the methylation status of *SIRT1* in paraffin-embedded tissue samples of oral squamous cell carcinoma (OSCC) obtained from BQ chewing and non-chewing patients and in tissue samples from healthy control subjects. In addition, we examined whether the hypermethylation of *SIRT1* followed by its transcriptional downregulation in the human gingival epithelial cells could be caused by arecoline, a major component of BQ. Furthermore, we investigated the methylation status of *SIRT1* in smear samples of macroscopically healthy buccal mucosa from subjects with a habit of BQ chewing.

**Results:**

*SIRT1* was significantly hypermethylated in tissue samples of OSCC from BQ chewers and non-chewers than in oral mucosa from healthy control subjects. Results also showed that the hypermethylation level of *SIRT1* was significantly higher in OSCC of patients with BQ chewing habits than in those of non-chewing habits (*p* < 0.05). Our in vitro model showed that hypermethylation is followed by downregulation of the transcriptional level of *SIRT1* (*p* < 0.05). The methylation levels of *SIRT1* in the smear samples obtained from BQ chewing individuals were significantly higher than those in the samples obtained from individuals that did not chew BQ. The duration of BQ chewing habits was correlated positively to the frequency of *SIRT1* hypermethylation (*p* < 0.05).

**Conclusions:**

Our results suggest that DNA hypermethylation of *SIRT1* is involved in the occurrence of oral cancer in BQ chewing patients and that hypermethylation in the oral mucosa of BQ chewers could be a predictive marker for the occurrence of malignant transformation. This is the first report that showed DNA hypermethylation in clinically healthy oral epithelium of BQ chewers. Our study shows evidence that DNA hypermethylation may be an early event of oral carcinogenesis prior to observable clinical changes.

## Background

The habit of betel quid (BQ) chewing is considered to be a significant risk factor for oral cancer and oral mucosal diseases in South and Southeast Asian countries [1–3]. BQ may be considered as any quid comprised of betel leaf and a combination of areca nut and slaked lime, with or without tobacco. Each component in BQ may individually, synergistically, and coordinately participate in carcinogenesis [2]. However, the underlying molecular mechanisms for the development of oral cancer remain unclear. Other environmental risk factors, such as tobacco and alcohol, can induce genetic and epigenetic modifications leading to oral cancer [4]. In addition to genetic modifications including mutations [1], DNA hypermethylation of tumor suppressor genes (TSGs) is a common epigenetic event observed in oral cancer [5], and environmental factors have been implicated in the hypermethylation of TSGs [4]. However, few studies have identified DNA hypermethylation in BQ chewing-related oral cancer and precancerous lesions [6–8]. Arecoline, a major alkaloid in areca nuts, is considered to be the most significant procarcinogen present in BQ [9]. Previous studies have demonstrated that arecoline may promote oral cancer by inducing transcriptional downregulation of TSGs [10, 11] and that this downregulation may be induced by DNA hypermethylation [12, 13]. However, the role of arecoline in DNA hypermethylation followed by downregulated transcriptional levels has not been clarified.

Sirtuins (SIRTs) are nicotinamide adenine dinucleotide (NAD^+^)-dependent histone deacetylases consisting of seven members (*SIRT1*–7) [14]. Each of the seven SIRTs has unique characteristics, functions, and localizations [15]. *SIRT1* was the first family member to be discovered and is well studied. Deregulated *SIRT1* expression was demonstrated previously in various human malignant diseases including oral cancer [16]. However, the physiological relevance of *SIRT1* in BQ-related oral cancer remains unexplored. BQ-related oral cancer is often preceded by the development of precancerous lesions, characterized by the disruption of epithelial integrity and, consequently, the transformation to invasive cancer [2]. Intriguingly, *SIRT1* has been identified as playing a role in the maintenance of epithelial integrity and contributing to the prevention of both the invasion and metastasis potential of the oral epithelium [17]. From these observations, we hypothesize that decreased *SIRT1* expression may occur in oral cancer induced by BQ chewing habit. Since the downregulated expression of *SIRT1* has been attributed to DNA hypermethylation [18], we hypothesize that DNA hypermethylation of *SIRT1* may be observed followed by its transcriptional downregulated expression in BQ chewing oral cancer patients.

In the present study, we analyzed the methylation status of *SIRT1* in paraffin-embedded tissue samples of oral squamous cell carcinoma (OSCC) obtained from BQ chewing and non-chewing patients and in tissues samples from healthy control subjects. In addition, we examined whether the hypermethylation of *SIRT1* followed by its transcriptional downregulation in the human gingival epithelial cells could be caused by arecoline, a major component of BQ. Furthermore, we investigated the methylation status of *SIRT1* in smear samples of macroscopically healthy buccal mucosa from subjects with a habit of BQ chewing.

## Results

### DNA methylation status of *SIRT1* in OSCC obtained from BQ chewing and non-chewing patients

Seventy-four patients were included in this study (39 males and 35 females). The patients were 24 BQ chewers with OSCC, 25 BQ non-chewers with OSCC, and 25 non-chewing healthy control subjects. Samples of OSCC of BQ chewers were obtained from Sri Lankan patients (*n* = 24), while samples of OSCC of BQ non-chewers (*n* = 25) and samples of healthy controls (*n* = 25) were obtained from Japanese subjects. The demographic data of the participants are listed in Table 1. Pearson’s chi-squared test revealed a significant difference in the gender ratio of tissue samples collected from BQ chewers with OSCC from non-chewers with OSCC and healthy control subjects. A significantly greater number of male patients was observed in the BQ chewers with OSCC than in BQ non-chewers with OSCC and healthy controls. A greater number of female subjects comprised the BQ non-chewers with OSCC group than in the BQ chewers OSCC and healthy controls groups. The levels of *SIRT1* hypermethylation was significantly altered in the OSCC from BQ chewers and non-chewers groups than in that from healthy controls. The hypermethylation levels were significantly higher in OSCC of BQ chewers compared to non-chewing OSCC patients (*p* < 0.05; Table 1).

**Table 1:** Characteristics of patients and tissue samples

	BQ chewers OSCC	BQ non-chewers OSCC	Healthy controls	Total	*p* value
Samples, *N* (%)	24 (32.4)	25 (37.8)	25 (37.8)	74 (100)	
Gender					0.005*
Male, *N* (%)	19 (79.2)	9 (36)	11 (44)	39 (52.7)	
Female, *N* (%)	5 (20.8)	16 (64)	14 (56)	35 (47.3)	
Age (years)	56.3 ± 14.2	61.5 ± 14.5	54.7 ± 16.3		0.253**
*SIRT1* DNA average methylation level	45.5 ± 15.0_a_	23.7 ± 14.0_b_	13.6 ± 9.8_c_		0.000**

Data are expressed as mean ± standard deviation (SD), values in italics represent statistical significance (*p* < 0.05), and subscripts that do not share the same in the row differ at *p* < 0.05 in the one-way ANOVA test

*BQ* betel quid, *OSCC* oral squamous cell carcinoma

*p value is calculated using Pearson’s chi-squared test

**p value is calculated using one-way ANOVA test

Furthermore, we examined the promoter methylation status of *SIRT1* in OSCC tissue samples obtained from BQ chewing and non-chewing patients, and oral mucosa samples from healthy control subjects. Multivariable regression analysis was conducted with Bonferroni adjusted *p* values using DNA methylation as a dependent variable, and age, sex, and groups (analysis I, BQ chewers vs healthy controls; analysis II, BQ non-chewers vs healthy controls; analysis III, BQ chewers vs BQ non-chewers) as independent variables. Analysis I investigated BQ chewing associated with OSCC, while analysis II investigated BQ non-chewing with OSCC. Analysis III investigated BQ chewing and non-chewing associated with OSCC. The results showed that *SIRT1* was significantly hypermethylated in tissue samples of OSCC from BQ chewers and non-chewers compared to oral mucosa from healthy control subjects. Results also showed that hypermethylation of *SIRT1* was significantly higher in OSCC from BQ chewing patients than in that from non-chewing patients (*p* < 0.05; Table 2).

**Table 2:** Multivariable regression analysis of SIRT1 DNA methylation level (total tissue samples)

Variables	*B*	Standard error	Beta	*t*	*p* value
Analysis I					
Age	0.102	0.123	0.076	0.833	1.000
Sex (male 1, female 2)	0.456	4.057	0.011	0.112	1.000
BQ chewers OSCC vs healthy controls	31.497	3.939	0.782	7.997	*0.000*
Analysis II					
Age	0.081	0.114	0.098	0.708	1.000
Sex	2.474	3.536	0.094	0.700	1.000
BQ non-chewers OSCC vs healthy controls	5.391	1.774	0.419	3.039	*0.012*
Analysis III					
Age	0.066	0.151	0.053	0.435	1.000
Sex	2.619	4.783	0.072	0.548	1.000
BQ chewers OSCC vs BQ non-chewers OSCC	21.005	4.806	0.587	4.370	*0.000*

Values in italics represent statistical significance (*p* < 0.05); *p* value is calculated using Bonferroni adjustment

*B* unstandardized coefficients, *Beta* standardized coefficients, *BQ* betel quid, *OSCC* oral squamous cell carcinoma

### The effects of arecoline on *SIRT1* DNA methylation, mRNA expression, and protein production

**Fig. 1:**
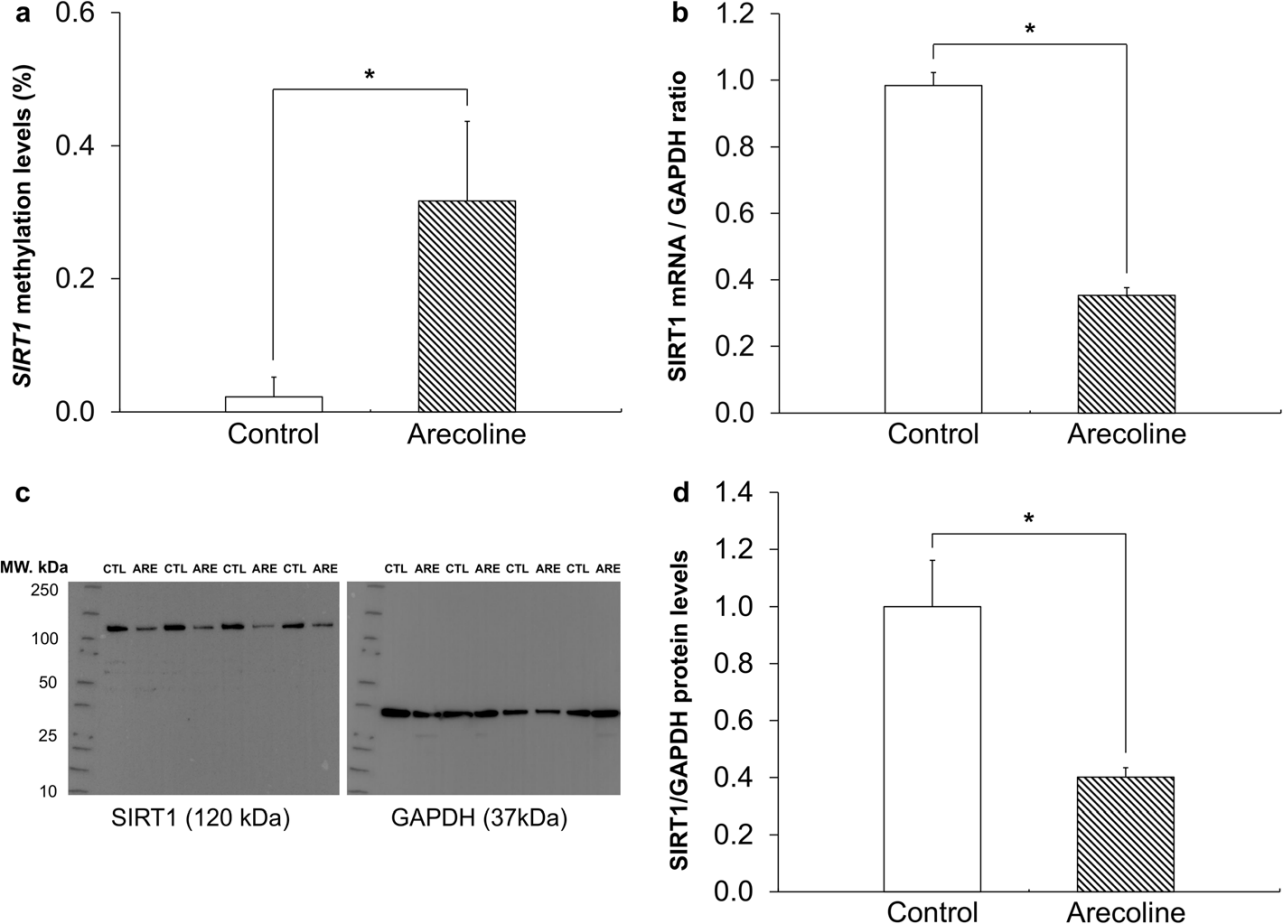
**a** The levels of *SIRT1* DNA methylation in cells treated with 50 μg/mL arecoline were significantly increased compared to that in control cells. **b** The expression levels of *SIRT1* mRNA in cells treated with arecoline at 50 μg/mL was significantly decreased compared to that in control cells. **c** The production of SIRT1 protein (bands of 120 kDa) was reduced by arecoline treatment compared to controls. As a control, the levels of GAPDH (bands of 37 kDa) were similar in all cells. **d** The ratio of intensities of SIRT1 to GAPDH in control cells was considered to be 100%. The ratio of intensities of SIRT1/GAPDH in control cells and arecoline-treated cells was 100 ± 16.2% and 40.1 ± 3.3%, respectively. The SIRT1 protein levels were reduced by arecoline treatment. Each experiment was performed in triplicate and results with *p* values of < 0.05 were considered to be statistically significant. CTL, control; ARE, arecoline

In order to confirm whether the hypermethylation of *SIRT1* caused by BQ chewing downregulates the transcriptional level of *SIRT1* in human gingival epithelial progenitors (HGEPs), HGEPs were stimulated with arecoline, a major component of BQ. The methylation level of *SIRT1* in cells treated with arecoline was significantly increased compared to that of control cells (*p* < 0.05; Fig. 1a). The expression levels of *SIRT1* mRNA in the cells treated with arecoline were significantly decreased compared to the control group (*p* < 0.05; Fig. 1b). To determine whether the mRNA expression levels of *SIRT1* relates to its protein production, further analysis was performed using western blotting to investigate the effects of arecoline on SIRT1 protein production. The protein levels of SIRT1 (bands of 120 kDa) were reduced by arecoline treatment in HGEP cells compared to that in controls. On the other hand, the protein levels of GAPDH (bands of 37 kDa) were the same in all cells (Fig. 1c). The ratio of intensities of SIRT1 to GAPDH (SIRT1/GAPDH) in controls was considered to be 100%. The ratio of intensities of SIRT1/GAPDH in control and arecoline HGEP cells were 100 ± 16.2% and 40.1 ± 3.3%, respectively. SIRT1 protein levels were reduced by arecoline in HGEP cells (*p* < 0.05; Fig. 1d). These results indicate that *SIRT1* mRNA transcription is suppressed by arecoline, resulting in decreased protein production in HGEPs. Together, these results demonstrate that DNA hypermethylation is involved in *SIRT1* transcriptional downregulation in HGEP cells following chronic stimulation with arecoline.

### Subject characteristics and profiling of *SIRT1* DNA methylation

A total of 70 adult participants were enrolled in the current study, including 14 males and 56 females. Based on the history of oral habits, the participants were classified into two groups: controls (45; healthy, non-chewers) and BQ chewers (25; healthy, chewers). The mean age of controls and BQ chewers was 35.5 ± 13.6 years and 39.0 ± 11.3 years, respectively. There were no significant differences observed in terms of gender and age to buccal smear samples of BQ chewers and non-chewers. The mean chewing years in the BQ chewers group were 7.3 ± 10.3 years. Detailed demographics of controls and BQ chewer groups are summarized in Table 3. Of the 25 BQ chewers, 5 (24%) has used BQ for 6 months or more, 14 (56%) chewed for 1–10 years, and 5 (20%) has used BQ for more than 10 years. The *SIRT1* DNA methylation levels in the buccal smear samples of BQ chewers were significantly higher than in that of non-chewers subjects (*p* < 0.05; Table 3).

**Table 3:** Characteristics of human participants and buccal smear samples

	Control (non-chewer)	Betel quid chewer	Total	*p* value
Samples, *N* (%)	45 (64.3)	25 (35.7)	70 (100)	
Gender				0.628*
Male, *N* (%)	9 (20)	5 (20)	14 (20)	
Female, *N* (%)	36 (80)	20 (80)	56 (80)	
Age (years)	35.5 ± 13.6	39.5 ± 12.0		0.200**
Duration of betel quid chewing habits				
≥ 6 months, *N* (%)	–	6 (24)	25 (35.7)	
1–10 years, *N* (%)	–	14 (56)		
> 10 years, *N* (%)	–	5 (20)		
Chewing years (mean ± SD)	–	7.3 ± 10.3		
*SIRT1* DNA average methylation level	4.0 ± 4.6	16.5 ± 23.7		0.001**

Data are expressed as mean ± standard deviation (SD); values in italics represent statistical significance (*p* < 0.05)

*p value is calculated using Pearson’s chi-square test

**p value is calculated using one-way ANOVA test

In this study, we investigated possible correlations between BQ chewing habits and the DNA methylation status of the *SIRT1* promoter region in oral mucosal epithelium. Multivariable regression analysis was conducted with Bonferroni adjusted *p* values using DNA methylation as a dependent variable, and age, sex, and BQ chewing habit as independent variables. The results showed that BQ chewing habit was the only significant predictor of DNA methylation (*p* < 0.05; Table 4 (A)). Further, among the BQ chewers group, multivariable regression analysis was conducted with Bonferroni adjusted *p* values using DNA methylation as a dependent variable, and age, sex, and chewing years as independent variables. It was revealed that the duration of chewing habit was significantly correlated to the levels of DNA methylation (*p < 0.05*; Table 4 (B)). These results demonstrate that BQ chewing and the duration of BQ chewing habits are positively correlated with DNA methylation frequency of the *SIRT1* gene of oral epithelia.

**Table 4:** Multivariable regression analysis of *SIRT1* DNA methylation level (buccal smear samples)

Variables	*B*	Standard error	Beta	*t*	*p* value
A. Total smear samples (*N* = 70)					
Age	0.219	0.134	0.183	1.630	0.324
Sex (male 1, female 2)	3.107	4.314	0.080	0.720	1.000
Betel quid chewers vs non-chewers	11.633	3.645	0.358	3.192	*0.007*
B. Betel quid chewer group (*N* = 25)					
Age	0.189	0.396	0.095	0.478	1.000
Sex	9.529	9.782	0.164	0.974	1.000
Chewing years	1.280	0.455	0.558	2.814	*0.031*

Values in italics represent statistical significance (*p* < 0.05); *p* value is calculated using Bonferroni adjustment

*B* unstandardized coefficients, *Beta* standardized coefficients

## Discussion

Results of the present study demonstrated that *SIRT1* in OSCC is hypermethylated and that the methylation levels were significantly higher in the OSCC of BQ chewers than in that of non-chewers. Our in vitro model showed that the hypermethylation is followed by downregulation of the transcriptional level of *SIRT1*. A higher level of methylation of *SIRT1* was observed in smear samples obtained from macroscopically healthy buccal mucosa in BQ chewers than in non-chewers. These results suggest that *SIRT1* is involved in the oral cancer caused by BQ chewing and that hypermethylation of *SIRT1* in the oral mucosa of BQ chewers may be a predictive marker for detecting early events in multistage carcinogenesis.

Although hypermethylation of *SIRT1* has been reported in several cancer tissues [19–21], this is the first demonstration of hypermethylation of *SIRT1* in OSCC. We confirmed the occurrence of *SIRT1* hypermethylation in OSCC of BQ chewers and non-chewers. We also found that the hypermethylation level of *SIRT1* was significantly higher in OSCC of patients with BQ chewing habits than in those of non-chewing habits. These results indicate that the DNA hypermethylation of *SIRT1* caused by BQ chewing is involved in BQ-related OSCC. It was not known whether the hypermethylation of *SIRT1* caused by BQ chewing is linked to *SIRT1* transcription. The extraction of RNA from paraffin-embedded tissue samples remains extremely challenging, and no consensus or standardized isolation method has been described [22]. Therefore, we employed an in vitro model of a daily BQ chewing habit that we showed previously to contain hypermethylated genes [23]. The cells were stimulated with arecoline, a major component of BQ, for a prolonged period according to our previous protocol [24]. We confirmed that significantly high level of methylation of *SIRT1* was observed followed by downregulated expression of *SIRT1* transcription and protein expression. This hypermethylation of *SIRT1* may cause the downregulated expression of *SIRT1* observed in OSCC. These results support previous findings suggesting *SIRT1* as a tumor suppressor [17, 25]. *SIRT1* has been reported to play a role in maintaining epithelial integrity by inducing the expression of epithelial-cadherin. Downregulation of *SIRT1* expression may weaken epithelial-epithelial interaction leading to malignant transformation of the epithelia [17]. The hypermethylation of *SIRT1* caused by arecoline in BQ chewers epithelium may be related to the instability of epithelial-epithelial interactions causing malignant transformation. It is still unknown how arecoline causes the hypermethylation of *SIRT1*. The promoter region of *SIRT1* possesses a potential regulator of epigenetic factors, methyl-CpG-binding protein 2 (MeCP2) [26]. MeCP2 has been shown to interact with DNA methyltransferase 1 (DNMT1) and recruits the latter to induce *SIRT1* promoter methylation [26]. Arecoline was previously documented to promote oral submucosal fibrosis and the progression to oral cancer through pathways involved in transforming growth factor-beta (TGF-β) production [2, 27]. TGF-β is likely to silence *SIRT1* epigenetically by inducing the MeCP2 expression, although other possible mechanisms cannot be ruled out [26]. These findings may provide an underlying molecular mechanism of the effect of arecoline on DNA hypermethylation. From these data, we hypothesized that the methylation level of *SIRT1* in healthy oral epithelium of BQ chewing subjects is higher than that of non-chewing subjects. We showed that the methylation level of *SIRT1* in smear samples obtained from macroscopically healthy buccal mucosa of BQ chewers is significantly higher than that in the samples of BQ non-chewers. The duration of chewing habits was correlated positively to the frequency of *SIRT1* hypermethylation in our data. This observation may support the previous paper that showed increasing the years of quid chewing habits was positively associated with oral cancer [28], wherein *SIRT1* hypermethylation may play an important role in the process of their development. Together, these findings indicate that DNA hypermethylation of *SIRT1* in epithelium of BQ chewers may be an early event involved in oral carcinogenesis. Previous reports confirmed DNA hypermethylation in precancerous lesions and oral cancer with the habits of BQ chewing [6, 7, 28]. However, no studies have shown alteration of DNA methylation in the macroscopically healthy epithelium of BQ chewers. To be the best of our knowledge, this is the first report that showed DNA hypermethylation in clinically healthy oral epithelium of BQ chewers. This result indicates that DNA hypermethylation may be caused by some carcinogen as an early event of carcinogenesis before their clinical changes. Therefore, examination of *SIRT1* hypermethylation, as well as other TSGs in smears of buccal mucosa, could be useful for the detection of early changes caused by BQ chewing habits.

Sampling buccal mucosa and saliva are the two most common non-invasive methods for genetic, epigenetic, and proteomic studies [29]. However, salivary ribonucleases rapidly degrade epithelial cell RNA during collection, and usable RNA has not been extracted from scrapings of buccal mucosa [30]. Therefore, DNA methylation analysis using buccal smear samples may be used as a molecular screen for oral cancer, particularly in areas with limited resources. A few previous studies investigated interactions between BQ chewing habits and DNA methylation using smear samples from buccal OSCC [8, 31]. However, our study is the first report that shows increased levels of DNA methylation in healthy buccal mucosa samples obtained from BQ chewers. Cigarette smoking and alcohol consumptions are other risk factors for oral cancer [32]. Those habits also cause alteration of DNA methylation [33, 34]. In fact, previous studies demonstrate clear evidence that development of oral cancer follows the same biological pathways irrespective of the source of carcinogenic exposure [35, 36]. Therefore, the hypermethylation of *SIRT1* may be a target for the prediction of oral carcinogenesis caused by those habits, as well as BQ chewing. Further investigations are needed to examine this hypothesis.

## Conclusions

In conclusion, our data demonstrate that DNA hypermethylation of *SIRT1* occurs in OSCC and normal oral mucosa obtained from BQ chewers and that the methylation status in buccal smear samples might be considered as an applicable routine oral screening procedure in high-risk populations, particularly in relation to BQ-induced oral cancers. Further studies are necessary to confirm our findings, which might lead to a better understanding of the molecular basis of oral carcinogenesis induced by various environmental exposures.

## Materials and methods

### Ethics statement

All participants in the study provided written informed consent, and the study was approved by the Institutional Review Boards of the Ethics Committee on Human Genetic Research at the Health Sciences University of Hokkaido, Japan (number # 2016-025), and the Ethical Committee at the University of Peradeniya, Sri Lanka (number # 7/2004).

### Tumor specimen and tissue collection

Oral squamous cell carcinoma (OSCC) and normal oral mucosa tissue samples were obtained from patients treated surgically. Twenty-four OSCC tissue samples were obtained from patients with BQ chewing habit in Sri Lanka. Twenty-five OSCC tissue samples were obtained from Japanese patients without BQ chewing habit, and 25 normal oral mucosae were obtained from individuals who underwent oral surgical intervention from 2008 to 2014 at the Health Sciences University of Hokkaido (HSUH) Hospital. The postsurgical tissue sections were formalin-fixed, processed, and paraffin-embedded following standard protocols. None of these patients received chemotherapy or radiotherapy prior to tumor resection. Data on patient demographics were retrieved from the archives of the Oral Medicine and Pathology Department at HSUH, Japan.

### Quantitative methylation-specific PCR

Genomic DNA was extracted from the tissue samples using DNeasy® Blood & Tissue Kit (Qiagen, Venlo, Netherlands), following the manufacturer’s instructions. The extracted DNA samples were treated with sodium bisulfite using the EpiTect® Plus Bisulfite Kits (Qiagen). DNA methylation of the *SIRT1* gene was analyzed using SYBR green-based quantitative methylation-specific PCR (qMSP). Two sets of primers were used: one for methylated and one for unmethylated DNA sequences [37]. The primers used for *SIRT1* gene were as follows (methylated, forward: GGCGAATTTGGTTGTATTATACG, reverse: GAACGAAAACTATTACGTCTACCG; unmethylated, forward: GGGGTGAATTTGGTTGTATTATATG, reverse: AAACAAAAACTATTACATCTACCACT). For PCR, the bisulfite-treated DNA template was mixed with KAPA SYBR FAST qPCR Kit and a pair of primers. The PCR conditions included initial incubation at 50 °C for 2 min, denaturing at 95 °C for 10 min, 50 cycles of denaturing at 95 °C for 15 s, and annealing at 58 °C for 1 min. After PCR amplification, a dissociation curve was generated to confirm the size of the PCR product. The percentage of DNA methylation in a sample was estimated using the following formula:

Methylated DNA $$ \left(\%\right)=\frac{M}{M+U}\times 100=\frac{1}{1+\frac{U}{M}}\times 100=\frac{1}{1+{2}^{\left(-\varDelta Ct\right)}} \times 100 $$ ,

where *M* is the copy number of methylated DNA, *U* is the copy number of unmethylated DNA, and *∆*Ct = Ct_*U*_–Ct_*M*_ [38]. Each experiment was performed in triplicate. Data are expressed as mean ± standard deviation (SD).

### Cell culture and arecoline exposure

**Fig. 2:**
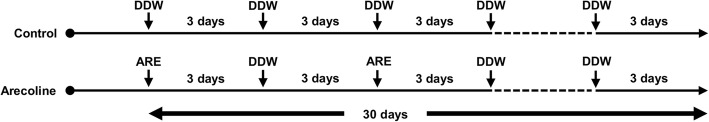
Flow chart of cell culture. Human gingival epithelium progenitors (HGEPs), cells were treated with arecoline at a concentration of 50 μg/mL. The culture media was replaced every 3 days, alternating media with and without arecoline for 30 days. Untreated samples were used as controls. DDW, double-distilled water; ARE, arecoline

Human gingival epithelial progenitors (HGEPs) and primary keratinocytes derived from healthy gingival epithelium were purchased from CELLnTEC Advanced Cell Systems (Basel, Switzerland) and cultured in CnT-Prime epithelial culture medium (CELLnTEC Advanced Cell Systems) at 37 °C in a humidified atmosphere of 95% air and 5% CO_2_. HGEPs were spread onto 100 mm tissue culture plates at a density of 4.0 × 10^4^ cells/mL. Arecoline (arecoline hydrobromide) was purchased from Sigma-Aldrich (St. Louis, MO). Following overnight incubation, the HGEPs were treated with arecoline at a concentration of 50 μg/mL. The concentration of arecoline used in this study was as described in previous experiments [24]. Briefly, arecoline at the concentration of 50 μg/mL had no cytotoxic effect on the cells stimulated, even for a prolonged period, the method of alternating between 3 days with 50 μg/mL of arecoline and 3 days without arecoline for 1 month was selected. The untreated samples were used as controls (Fig. 2).

### Quantitative methylation-specific PCR

Genomic DNA was extracted from the HGEPs using DNeasy® Blood & Tissue Kit (Qiagen), following the manufacturer’s instructions. The extracted DNA samples were treated with sodium bisulfite using the EpiTect® Plus Bisulfite Kits (Qiagen), and DNA methylation of the *SIRT1* gene was performed using the method described previously [38]. Each experiment was performed in triplicate. Data are expressed as mean ± SE of the DNA methylation.

### Real-time quantitative reverse-transcription PCR

Total RNA was extracted from the HGEPs using the RNeasy® Mini Kit (Qiagen) following the manufacturer’s instructions. Total RNA was reverse transcribed into cDNA using a ReverTra Ace® qPCR RT Master Mix (Toyobo, Osaka, Japan). The cDNA levels were measured using the LightCycler® Nano System (Roche Diagnostics, Basel, Switzerland). Two sets of primer were used (*SIRT1*, forward: GCGATTGGGTACCGAGATAA, reverse: TTGCATGTGAGGCTCTATCC; *GAPDH*, forward: GTGAAGGTCGGAGTCAAC, reverse: GTTGAGGTCAATGAAGGG) [39, 40]. For qRT-PCR, cDNA was mixed with KAPA SYBR FAST qPCR Kit (Nippon Genetics, Tokyo, Japan) and a pair of primers. The PCR conditions included initial incubation at 50 °C for 2 min, denaturing at 95 °C for 10 min, 40 cycles of denaturing at 95 °C for 15 s, and annealing at 60 °C for 1 min. The relative expression of each mRNA was calculated as the Ct (the value obtained by subtracting the Ct value of the GAPDH mRNA from the Ct value of the target mRNA) using the ∆∆Cq method [41]. Specifically, the amount of target mRNA relative to GAPDH mRNA is expressed as 2^−(∆Ct)^. Each experiment was performed in triplicate. Data are expressed as mean ± standard error (SE) of the ratio of the target mRNA to GAPDH mRNA.

### Western blotting analysis

Proteins were extracted from the HGEPs using lysis buffer [50 mM Tris HCL, pH 7.5; 10 mM EDTA, pH 7.5; 165 mM NaCl; 10 mM NaF; 1% Nonidet P-40; 1 mM PMSF; 1 mM NaVO_3_; 10 μg/mL leupeptin; and 10 μg/mL aprotinin]. The lysis reaction was carried out for 1 h at 4 °C. The samples were centrifuged at 15,000 rpm for 30 min at 4 °C, and the supernatant was used as sample. Protein concentration was quantified by Lowry’s protein assay. Fifteen micrograms of protein samples were used for western blotting analysis. Sodium dodecyl sulfate-polyacrylamide gel electrophoresis (SDS-PAGE) was carried out in pre-cast gels (4–20% gradient of polyacrylamide; Mini-PROTEAN TGX Gels; Bio-Rad, Hercules, CA, USA). After electrophoresis, gels were transferred electrophoretically onto polyvinylidene difluoride membranes (Immobilon-P; Millipore, Bedford, MA, USA) and blocked for 1 h with Tris-buffered saline (TBS) containing 5% skimmed milk. Blocked membranes were washed twice with TBS containing 0.05% Tween-20 solution.

The following primary antibodies were used: mouse monoclonal antibody against SIRT1 (dilution 1:1000, #ab110304; Abcam, Cambridge, MA, USA) and rabbit monoclonal antibody against GAPDH (dilution 1:5000, #CST5174; Cell Signaling Technology, Danvers, Massachusetts, USA). Membranes were incubated with the primary antibody overnight at 4 °C, washed three times with TBS containing 0.05% Tween-20 solution, and incubated with a horseradish peroxidase-conjugated secondary antibody (dilution 1:10,000; Jackson Immuno-Research Laboratories Inc., West Grove, PA, USA) for 1 h at room temperature. Bands of SIRT1 and GAPDH were visualized by the enhanced chemiluminescence system (Clarity Max™ Western ECL Substrate; Bio-Rad) and LuminoGraph I (ATTO Corporation, Tokyo, Japan) and recorded using ImageSaver6 software (ATTO Corporation). Expression levels of SIRT1 and GAPDH in cells treated with or without arecoline were quantified by analyzing the intensity of each band using CS Analyzer4 software (ATTO Corporation). Each experiment was performed in triplicate. Data are expressed as mean ± SE of the ratio of the target protein to GAPDH protein.

### Buccal smear samples and clinicopathological data collection

Prior to sample collection, the nature of the study was fully explained to all participants. Information obtained from the interview included socioeconomic and demographic characteristics; personal and family histories; risk factors for oral cancer, such as lifestyle, alcohol drinking, tobacco smoking, and BQ chewing including its frequency; and the added use of betel leaf, areca nut, slaked lime, and tobacco. Clinic attendees of at least 20 years of age and with the ability to complete the questionnaires by an interview and clinical oral examinations were eligible to participate. The presence of oral mucosal lesions was evaluated and documented by a registered dentist, based on the recommendations of the World Health Organization [42]. Participants having oral mucosal lesions or any systemic disorders (such as diabetes, immune-compromised, or genetic diseases) were excluded from this study. A convenience sample of 70 study subjects was recruited and classified into two groups: controls (45 healthy, non-chewers) and BQ chewers (25 healthy, chewers). Both the controls and BQ chewer groups were non-smokers and non-drinkers. Subjects who had chewed one BQ per day for at least 6 months were considered as chewers.

For oral cancer screenings, samples for biomarker testing should be easily available. Thus, the present study used samples obtained by buccal smear, which is noninvasive and easy to perform and which may assist in screening for oral cancers, particularly in areas with limited resources. Buccal smear samples were collected by research staff according to the manufacturer’s instructions (Qiagen). Briefly, the participants were restricted in eating and drinking for 30 min prior to collection, and it was verified that the participant’s mouth was empty. The swab sticks were removed from the package carefully to avoid contaminating the tip of the swab with gloves or against any surface. The swab was firmly rubbed and rotated along the inside of the cheek for 5–10 times, and to ensure that the entire tip was in contact with the cheek, this step was repeated on the other cheek. The swab stick was removed from the mouth, being careful not to touch swab tips with any other surface such as teeth, lips, or other surfaces. The swab was placed directly into the tube containing the DNA stabilizing reagent Gentra® Puregene® buccal cell kits (Qiagen), and the tube was labeled with identifying information. Samples were stored at − 20 °C until shipment on ice to HSUH for testing.

### Quantitative methylation-specific PCR

Genomic DNA was extracted from the buccal smear samples using Gentra® Puregene® buccal cell kits (Qiagen), following the manufacturer’s instructions. The extracted DNA samples were treated with sodium bisulfite using the EpiTect® Plus Bisulfite Kits (Qiagen), and DNA methylation of the *SIRT1* gene was performed using the methods described previously [38]. Each experiment was performed in triplicate. Data are expressed as mean ± SD of the DNA methylation.

### Statistical analysis

Statistical analysis was performed on a database using IBM SPSS Statistics 23 (IBM, Armonk, NY). Pearson’s chi-squared test was used to analyze gender differences in groups of tissue samples (BQ chewers OSCC, BQ non-chewers OSCC, and healthy controls) and in buccal smear samples (control and BQ chewer). One-way ANOVA test was performed to analyze the age and DNA methylation level differences in tissue samples and buccal smear sample subjects. Multivariable regression analysis was performed with Bonferroni adjusted *p* values for multiple comparisons in tissue samples of OSCC and healthy control subjects, and buccal smear samples of BQ chewers and non-chewers subjects. Mann-Whitney *U* test was performed between groups of arecoline-treated and untreated samples. Results with *p* values of < 0.05 were considered to be statistically significant.

## Data Availability

All data generated or analyzed during this study are included in this published article.

## Abbreviations

BQ: Betel quid
DNMT1: DNA methyltransferase 1
MeCP2: Methyl-CpG-binding protein 2
OSCC: Oral squamous cell carcinoma
SIRT1: Sirtuin 1
SIRTs: Sirtuins
TGF-β: Transforming growth factor-beta
TSGs: Tumor suppressor genes
